# Tracheobronchomalacia in the patients with treatment-resistant severe asthma: case reports

**DOI:** 10.1186/2045-7022-3-S1-P23

**Published:** 2013-05-03

**Authors:** Sawad Boonpiyathad, Atik Sangsapaviliya

**Affiliations:** 1 Pramongkutklao Hospital, Department of Medicine, Thailand

## Background

Tracheobronchomalacia is defined as the condition that the airway lumen is 50 percent narrower than normal. The acquired tracheobronchomalacia usually occurs in adults, however this condition is not usually found in asthmatic patients.

## Purpose

We report two cases of the elderly patients who have severe persistent asthma that cannot be controlled with full asthma medication.

## Case reports

Case 1. 70 years old woman, with history of severe persistent asthma for 10 years was referred to our allergy clinic. She could not control her asthma and asthmatic attack always happened at night, so it was worse when she slept. She was treated with fluticasone/salmeterol accuhaler (250/50 mcg) 2 puffs twice daily, montelukast (10 mg), theophylline (200 mg), procaterol (50 mcg) and salbutamal evohaler 2-3 times daily. Pulmonary function test showed moderate restrictive lung disease, FEV1 59%. Additionally, chest CT scans detected collapse of trachea at posterior wall. Afterwards we treated tracheomalacia by continuous positive airway pressure at night. As a result, her asthma symptoms have been improving. Case 2. 72 years old woman, with history of severe persistent asthma for 15 years, was partly controlled (asthma controlled test score 20 and peak expiratory flow rate 180 l/min) with asthma medication such as fluticasone/salmeterol accuhaler (500/50 mcg) 2 puffs twice daily, montelukast (10 mg), theophylline (200 mg), tiotropium (18 mcg), salbutamal evohaler 4-5 times and omalizumab (300 mg) every two weeks. 3 months before investigating this case, her asthma could not be controlled (ACT score 7 and PEFR 100 l/min) despite she was treated by the oral corticosteroid (20 - 30 mg) every day. Her chest CT scans were normal, but the bronchoscope found bronchomalacia at her right and left bronchus of lower lungs.

## Conclusion

We reported 2 cases of tracheobronchomalacia that found in the patients with severe persistent asthma. Before diagnosis of refractory asthma, it is important to consider and exclude other diseases such as tracheobronchomalacia particularly in aged.

